# Mapping the amplitude and phase of dissolved ^129^Xe red blood cell signal oscillations with keyhole spectroscopic lung imaging

**DOI:** 10.1002/mrm.30296

**Published:** 2024-10-18

**Authors:** Jemima H. Pilgrim‐Morris, Guilhem J. Collier, Mika Takigawa, Scarlett Strickland, Roger Thompson, Graham Norquay, Neil J. Stewart, Jim M. Wild

**Affiliations:** ^1^ POLARIS, Section of Medical Imaging and Technologies, Division of Clinical Medicine, School of Medicine and Population Health University of Sheffield Sheffield UK; ^2^ Insigneo Institute University of Sheffield Sheffield UK; ^3^ Biomedical Research Centre University of Sheffield Sheffield UK; ^4^ Sheffield Teaching Hospitals Sheffield UK

**Keywords:** cardiogenic oscillations, hyperpolarized ^129^Xe, lung MRI, pulmonary microvasculature

## Abstract

**Purpose:**

To assess the regional amplitude and phase of dissolved ^129^Xe red blood cell (RBC) signal oscillations in the lung vasculature with keyhole spectroscopic imaging and to compare with previous methodology, which does not account for oscillation phase.

**Methods:**

^129^Xe gas transfer was measured with a four‐echo 3D radial spectroscopic imaging sequence. Keyhole reconstruction‐based RBC signal oscillation amplitude mapping was applied retrospectively to data acquired from 28 healthy volunteers, 4 chronic thromboembolic pulmonary hypertension (CTEPH) patients, and 5 patients who were hospitalized due to COVID‐19 pneumonia and had residual lung abnormalities. Using a sliding window keyhole reconstruction, maps of RBC oscillation amplitude were corrected for regional phase difference. Repeatability of the phase‐adjusted oscillation amplitude was assessed in 8 healthy volunteers across three scans.

**Results:**

With sliding window keyhole reconstruction, regional phase differences were observed in the RBC signal oscillations: mean phase = (0.27 ± 0.19) rad in healthy volunteers, (0.24 ± 0.13) rad in CTEPH patients, and (0.33 ± 0.19) rad in patients with post‐COVID‐19 residual lung abnormality. The oscillation amplitude and phase maps were more heterogeneous (i.e., they showed increased coefficient of variation) for the CTEPH patients. The RBC oscillation amplitude was repeatable, and the mean three‐scan coefficient of variation was smaller when the phase adjustment was made (0.07 ± 0.04 compared with 0.16 ± 0.05).

**Conclusion:**

Sliding window keyhole reconstruction of radial dissolved ^129^Xe imaging reveals regional phase differences in the RBC oscillations, which are not captured when performing two phase keyhole reconstruction. This regional phase information may reflect the hemodynamic effect of the cardiac pulse wave in the pulmonary microvasculature.

## 1 INTRODUCTION

Hyperpolarized (HP) ^129^Xe MRI is a powerful tool for functional lung imaging, providing regional information about lung ventilation, microstructure, and gas exchange.^1^ ^129^Xe is soluble in the alveolar parenchymal tissue, capillary blood plasma, and red blood cells (RBC), giving rise to dissolved ^129^Xe signals distinct from the gaseous ^129^Xe resonance, with a chemical shift of about 200 ppm. The dissolved ^129^Xe signals comprise a tissue‐plasma (collectively “membrane” [M]) peak and an RBC peak, which occur at approximately 197 ppm and 218 ppm, respectively.^2^ Spectral encoding of the dissolved compartment gives HP ^129^Xe MRI unique sensitivity for measuring alveolar‐to‐capillary gas diffusion in diseases such as interstitial lung disease (ILD), asthma, and chronic obstructive lung disease (COPD).^2^, ^3^, ^4^, ^5^, ^6^, ^7^, ^8^, ^9^

In dissolved‐phase ^129^Xe lung spectroscopy, the M and RBC signals decay over the 10–15‐s duration breath hold, due to RF pulse‐induced depolarization and T_1_ relaxation of the gas‐phase signal, which acts as a magnetization “reservoir” replenishing dissolved‐phase signal (T_1_ ˜ 20 s).^10^ Additionally, the RBC signal is periodically modulated by the heartbeat frequency^11^, ^12^, ^13^; these oscillations originate from changes in the capillary blood volume over the cardiac cycle.^6^ The underlying ^129^Xe RBC signal is also dependent on the diffusion rate of ^129^Xe across the alveolar membrane and the hematocrit within the alveolar capillary bed.^2^, ^14^ The cardiogenic RBC oscillations revealed in ^129^Xe spectroscopy provide a potential means to monitor blood flow in the pulmonary microvasculature, which is often affected by chronic lung disease.^15^, ^16^ The ^129^Xe spectroscopy‐derived RBC oscillation has been shown to be sensitive to disease state: the oscillation amplitude is increased in idiopathic pulmonary fibrosis (IPF), nonspecific interstitial pneumonia, and left heart failure patients but decreased in COPD and pulmonary arterial hypertension patients when compared with healthy subjects.^3^, ^6^, ^9^, ^17^, ^18^ Furthermore, the amplitude of the RBC oscillations may be able to differentiate between pulmonary hypertension subtypes.^18^

Imaging microvascular function directly is difficult due to the small vessel size and the effects of cardiac and respiratory motion. Larger pulmonary blood vessels' form and function can be imaged using CT, MRI and echocardiography, whereas microvascular perfusion can be deduced indirectly from dynamic contrast‐enhanced MRI.^16^, ^19^, ^20^ However, unlike dissolved ^129^Xe spectroscopy, these techniques do not capture the gas exchange dynamics in the pulmonary capillary bed. Recently, Niedbalski et al. proposed a technique to spatially resolve the ^129^Xe RBC oscillations.^21^ The authors used a 3D radial k‐space trajectory and used the inherent oversampling at the center of k‐space (k_0_) to obtain dynamic signal information via a postacquisition keyhole reconstruction.^22^ By binning the k_0_ data according to RBC signal amplitude, reconstructing images from the radial spokes in “low” and “high” bins, and finding the difference between the resulting “low” and “high” keyhole images, regional RBC signal oscillations were mapped in a cohort of healthy volunteers, patients with IPF, and patients with pulmonary arterial hypertension. This method has since been further optimized using digital phantom simulations by Lu et al.^23^ and applied to patients with chronic thromboembolic pulmonary hypertension (CTEPH).

To separate the RBC and M signals, a 1‐point Dixon spectroscopic imaging approach has been used.^2^, ^24^ However, the 90° phase shift between the RBC and M signals evolves during readout, leading to image blurring. An alternative spectroscopic imaging method to differentiate the dissolved‐phase signal is a multipoint acquisition with^5^, ^25^ or without^9^, ^26^ the IDEAL (iterative decomposition of water and fat with echo asymmetry and least‐squares estimation).^27^ Collier et al. implemented a 4‐point flyback 3D radial sequence, which has been shown to detect gas exchange impairment in patients with IPF, asthma, COPD, and COVID‐19.^9^, ^26^, ^28^, ^29^, ^30^, ^31^, ^32^

In this work, we adapted the keyhole oscillation mapping method from Niedbalski et al.^21^ to spatially resolve the RBC oscillations in dissolved‐phase ^129^Xe lung spectroscopic images acquired using a multipoint acquisition.^26^ This method was then applied to data from 28 healthy volunteers, 4 CTEPH patients, and 5 patients who were hospitalized due to COVID‐19 pneumonia. In previous work,^21^, ^23^ the regional RBC oscillations were assumed to be in phase across the lung, and only two keyhole images, corresponding to average maximum and minimum RBC signal, were reconstructed. In this work, we hypothesize that the phase of the RBC oscillation is also spatially dependent. We show that the previous assumption of constant phase leads to a reduced and sometimes negative local RBC oscillation amplitude. To address this, we introduce a “sliding window” (SW) technique to reconstruct additional keyhole projections, to map not only the amplitude, but also the phase of the regional RBC oscillations. We present the quantitative mapping of this phase as a novel means to probe the effects of the cardiac pulse wave in the pulmonary capillary bed.

## 2 METHODS

### 2.1 Subject details

Data from healthy volunteers and patients from previous studies were retrospectively analyzed in this work.^28^ The subject cohort consisted of 28 healthy volunteers, 4 CTEPH patients, and 5 subjects who were imaged at either 6 or 12 months following hospitalization due to COVID‐19 pneumonia. All CTEPH patients were identified as having precapillary disease via right‐heart catheterization; clinical metrics including mean pulmonary arterial pressure and pulmonary vascular resistance can be found in Table S1. The patients hospitalized with COVID‐19 had residual lung abnormalities present at 12 weeks after hospital discharge, identified on CT imaging by a chest radiologist, and so are referred to as “post‐COVID‐19 residual lung abnormality” (PC‐RLA). Follow‐up CTs (mean time since discharge: 6.6 ± 2.7 months) identified ground glass opacities in 5 of 5 subjects, reticulation in 4 of 5 subjects, and fibrotic‐like changes in 2 of 5 subjects. All study protocols were approved by the National Research Ethics Committee (IRAS ref 265 997 for the PC‐RLA patients and STH18877 local ethics and governance for the CTEPH patients).

### 2.2 MRI acquisition

Imaging was performed on 1.5T whole‐body GE HDx (*n* = 29) and 1.5T GE Artist (*n* = 8) clinical scanners using a flexible ^129^Xe transmit‐receive vest coil and ^129^Xe polarized to about 30% with a custom spin exchange optical pumping polarizer.^33^ Subjects inhaled doses of 86% enriched ^129^Xe (0.8–1.0 L, depending on height) from a Tedlar bag, starting from functional residual capacity, and were imaged using a four‐echo 3D radial spectroscopic sequence^26^ over a 14‐s breath hold. A total of 934 radial spokes were acquired, with 13 samples per echo (30% oversampling in the readout direction). The bandwidth was 31.25 kHz, the voxel size was 2 cm^3^ with a FOV of 40 cm, and images were reconstructed to a 32^3^ matrix size. The dissolved‐phase ^129^Xe resonance was excited using a 1.2‐ms‐duration frequency‐selective RF pulse centered on the ^129^Xe M resonance. The TR and flip angle used were 15 ms and 22°, respectively (TR_90,equiv_ ˜ 200 ms).^34^ At the start of the sequence, 20 dummy RF pulses were used to both deplete the downstream signal from ^129^Xe in the pulmonary veins and acquire calibration spectra.

To assess the interscan and intrascan repeatability of the proposed RBC oscillation phase‐mapping method, 8 of the healthy volunteers (3 female, 5 male, ages 23–41) underwent additional imaging (once in the morning and once in the afternoon, with 3.5–5 h between the two sessions). In one of the sessions, the volunteers were scanned twice, with approximately 5 min between the scans.

### 2.3 Data analysis

Image preprocessing, reconstruction, and analysis were carried out in *MATLAB* (version 2022a; MathWorks, Natick, MA). The calibration spectra were used to estimate subject‐specific resonant frequencies and T_2_* of the ^129^Xe dissolved in the alveolar airspace, membrane, and capillaries. This was done with a triple Lorentzian fit in the frequency domain.^35^ The gas, RBC, and M resonances were separated in k‐space using a matrix inversion and prior knowledge of the chemical shifts and T_2_* of the resonances obtained from the calibration spectra.^26^, ^36^ The 3D radial reconstruction was performed using the optimal Kaiser‐Bessel convolution formula.^37^

**FIGURE 1:**
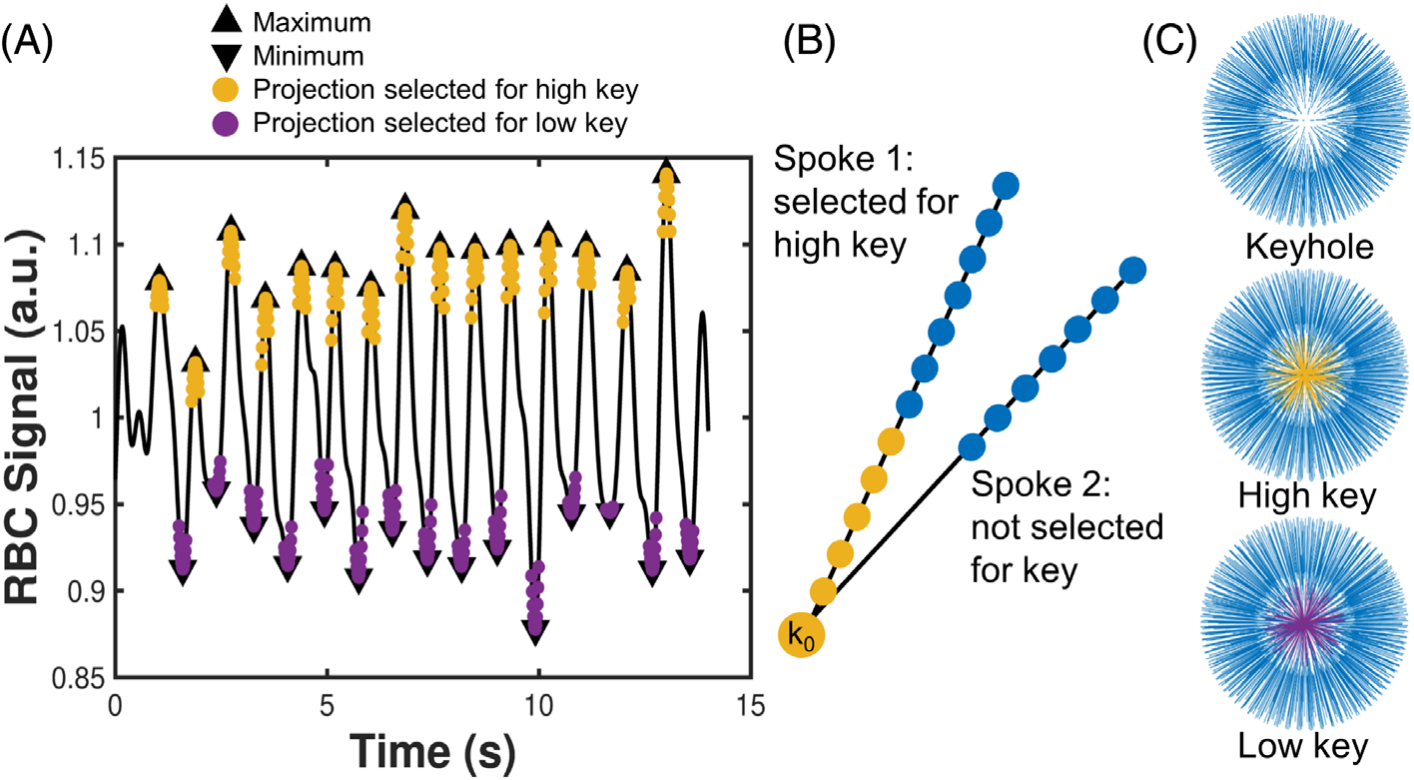
“Two‐Key” red blood cell (RBC) oscillation mapping. (A) A peak detection algorithm is applied to the detrended, normalized, and filtered k_0_ RBC signal (*black line*) to identify the maxima and minima (*black triangles*). Each point corresponds to the k_0_ signal for a given spoke in the 3D radial trajectory. The projections adjacent to the extrema are selected for the high key (*yellow*) or low key (*purple*). (B) The high‐frequency data (points 7–13 along the spoke, *shown in blue*) of every projection are included in the keyhole. Points 1 to 6 are only included for projections that have been selected for the key (*yellow points*). (C) The 3D radial k‐space data for the keyhole alone and the keyhole plus the high key, and the low key.

The *global* RBC oscillation amplitude was evaluated as follows (Figure S1). The k_0_ signal from the spectrally reconstructed membrane k‐space was normalized by its mean value and fit to a biexponential decay function. The RBC k_0_ signal was also normalized by its mean value and, to help detect the oscillations, was corrected for RF and T_1_ decay by multiplying by the inverse of the membrane k_0_ signal fit. A band‐pass filter of 0.5–2.5 Hz was used to smooth the signal, and a peak detection algorithm was used to identify the minima and maxima (black triangles in Figure 1A). The mean k_0_ peak‐to‐peak amplitude of the oscillations ($\alpha_{k0}$) was found from the difference between the mean of the maxima and minima in the first approximate 7 s of the breath hold, where the SNR was highest, multiplied by 100%. Heart rate was estimated from the frequency of these oscillations. The first second of acquisition (˜60 projections) was generally excluded from the analysis due to transient behavior. In some cases, where SNR decreased at the end of the data acquisition period such that the final oscillations became indistinguishable from noise, the last second of data acquisition was also excluded.

We define the RBC signal (after correction for RF and T_1_ decay) at position r=(x,y,z) and time t as follows:

(1) $$S\left(r,t\right)=\frac{\alpha \left(r\right)}{2}f\left(\omega t+\phi \left(r\right)\right),$$

where α(r) is the spatially dependent peak‐to‐peak ^129^Xe RBC signal amplitude, and f(ωt+ϕ(r)) is a periodic function with angular frequency ω (heartbeat frequency) and spatially dependent phase ϕ(r). To map the RBC oscillations *regionally*, two keyhole reconstruction methods were implemented and compared.

#### 2.3.1 Method 1: “Two‐Key” RBC oscillation mapping

The RBC oscillation maps were found from the difference in signal amplitude in the images corresponding to low and high keyhole data, as described in Niedbalski et al.^21^ After preprocessing with the steps described previously, the k_0_ projections adjacent to the minima and maxima were binned into low (purple circles in Figure 1A) and high (yellow circles in Figure 1A). The number of projections in each bin was approximately 20% of the total number of projections, and a keyhole radius of 6 points was used, as shown in Figure 1B. These selection criteria were chosen to maximize the radius of the key and therefore the oscillation mapping fidelity, while minimizing undersampling. The keys were then inserted separately into the high‐frequency “keyhole” data using the last 7 points from all spokes (blue in Figure 1B,C). To account for the fact that the k‐space sampling was no longer uniform, an iterative numerical density compensation function was used.^38^ Chemical shift separation and image reconstruction were then carried out for both the high and low keyhole data, resulting in two sets of ^129^Xe images corresponding to the “high” and “low” RBC signal ($S_{\text{high}}$ and $S_{\mathrm{low}}$, respectively). The Two‐Key RBC oscillation amplitude ($\alpha_{2-\mathrm{Key}}$) map was calculated from the pixelwise difference between $S_{\text{high}}$ and $S_{\mathrm{low}}$, divided by their mean and multiplied by 100%. This normalization ensures that the oscillation amplitude is dimensionless, that the amplitude at each pixel is normalized by its mean value, and that the maps are normalized for regional coil sensitivity that is intrinsic to both denominator and numerator.

#### 2.3.2 Method 2: Sliding window RBC oscillation phase mapping

**FIGURE 2:**
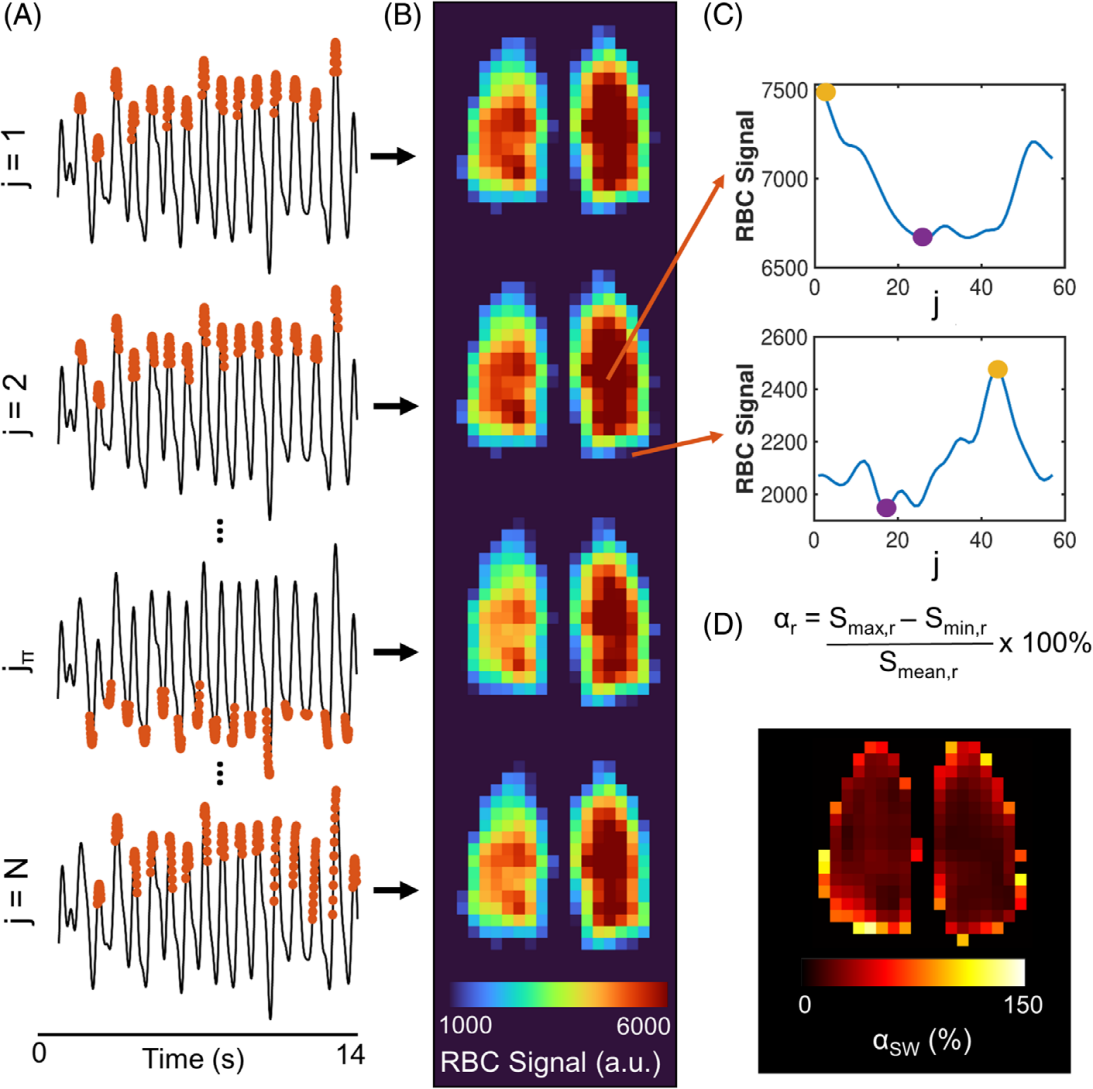
Overview of the “sliding window” (SW) red blood cell (RBC) oscillation phase‐mapping method. (A) The k_0_ RBC signal maxima are identified using a peak finding algorithm, and the adjacent projections (*red*) are included in the first key (*j* = 1), which is combined with the high‐frequency k‐space data in a keyhole reconstruction. The chosen projections are then stepped forward by one to form the second key (*j* = 2) and produce the second keyhole image, and then again for *j* = 3 to produce a third image, and so on. The value of *j*_π_ corresponds to the key where the chosen projections are approximately in antiphase with the projections from *j* = 1 (i.e., distributed around the k_0_ minima). *j* = *N* is the final key and should correspond to the projections from *j* = 1 shifted by one step to the left; however, this is generally not the case because of the unequal number of projections per wave cycle. (B) Keyhole images are produced for each key. (C) For every pixel, the minimum (*purple circle*) and maximum (*yellow circle*) RBC signal values as a function of key number (*j*) are found. (D) The oscillation amplitude at each pixel ($\alpha_{r}$) is found from the difference between the maximum and minimum RBC signal value across all keyhole images ($S_{\max}$ and $S_{\min}$), normalized by the mean value across all keyhole images ($S_{\text{mean}}$) and multiplied by 100%. This results in a phase‐corrected α map.

To account for regional phase differences in the RBC signal oscillation, a SW keyhole reconstruction was implemented. The k_0_ maxima were the same as with the Two‐Key method and the neighboring projections binned as before. Then, multiple keyhole reconstructions were performed, with the chosen projections stepped forward by one projection per reconstruction. Figure 2A shows the k_0_ projections chosen for four of the keyhole reconstructions, and Figure 2B shows the corresponding keyhole images for a central slice of the lung. The total number of keyhole reconstructions, *N*, was determined by the mean number of projections between adjacent maxima, such that one cardiac cycle was sampled.

The regional RBC signal formed from each keyhole reconstruction ($S_{\left(r,j\right)}$) was smoothed using a Gaussian‐weighted moving average filter with a window length of 10. For each pixel, the maximum and minimum signals across the *N* reconstructed images, referred to as $S_{\max}$ and $S_{\min}$, were found (Figure 2C). The SW oscillation amplitude ($\alpha_{\mathrm{SW}}$) was then calculated from the difference between $S_{\max}$ and $S_{\min}$, normalized by the mean signal of all *N* keyhole images ($S_{\text{mean}}$) and multiplied by 100% (Figure 2D). The index, *j*, of the keyhole image where $S_{\max}$ was found, denoted as *j*_max_, provides information about ϕ(r). If *j*_max_ = 1, then the oscillation is in phase with the global k_0_ oscillation, and ϕ=0. When ϕ=π, this signifies that the k_0_ projections selected by the SW are π out of phase with the k_0_ projections selected for the *j* = 1 keyhole image. By converting *j*_max_ to ϕ, the regional phase differences of the RBC signal oscillation can be quantified and visualized.

A graphical summary of how $\alpha_{k0}$, $\alpha_{2-\mathrm{Key}}$, $\alpha_{\mathrm{SW}}$, and ϕ are calculated is provided in Figure S2.

### 2.4 Image analysis

Reconstructed images were masked by applying a noise threshold to the membrane signal images. Regions of interest were created to analyze RBC oscillation differences in the left, right, upper, lower, anterior, posterior, central, and peripheral lung. The average $\alpha_{2-\mathrm{Key}}$, $\alpha_{\mathrm{SW}}$, and ϕ within the whole lung mask and eight regional masks were calculated for each subject.

### 2.5 Statistical methods

Statistical analysis was performed using RStudio (version 2023.03.1; R version 4.3.0). Normality of variables was determined with Shapiro–Wilk normality tests. Correlations among $\alpha_{k0}$, $\alpha_{2-\mathrm{Key}}$, $\alpha_{\mathrm{SW}}$, and heart rate were assessed using the Pearson correlation coefficient for normally distributed variables and Spearman's correlation coefficient for nonnormal variables. Differences between variables were tested with paired Student's t‐tests or Wilcoxon signed‐rank tests. Non‐parametric Kruskal‐Wallis tests were used to provide a preliminary indication of differences among the healthy, CTEPH, and PC‐RLA cohorts. These were followed by post hoc Dunn tests with a Benjamin‐Hochberg multiple comparisons correction. A significance level of *p* < 0.05 was used for all tests.

To assess repeatability, the following metrics were used: bias (mean difference between two scans), % difference (mean absolute percentage difference between two scans), and coefficient of variation (CV; across all three scans).

## 3 RESULTS

To ensure clear RBC k_0_ oscillations, only subjects with an RBC image SNR of above 4.5 were selected for this work. Cases with an SNR of between 4.5 and 5.5 (i.e., on the borderline of the Rose criterion^39^) were visually inspected for discernible RBC k_0_ oscillations before inclusion. This cutoff was chosen empirically based on preliminary analysis of the relationship between RBC SNR and the percentage difference between $\alpha_{k0}$ and $\alpha_{2-\mathrm{Key}}$ in healthy volunteers. As a result, 28 of 42 healthy data sets, 4 of 5 CTEPH, and 5 of 7 PC‐RLA patient data sets were included in this work. In this cohort of 37 subjects, keyhole RBC images were reconstructed for low and high RBC k_0_ signal and for each key of the SW method with minimal undersampling. Across all (healthy) subjects, the median of the maximum value of the iterative density compensation function was 1.46. The total keyhole mapping reconstruction and processing time was about 5 min for both methods.

**FIGURE 3:**
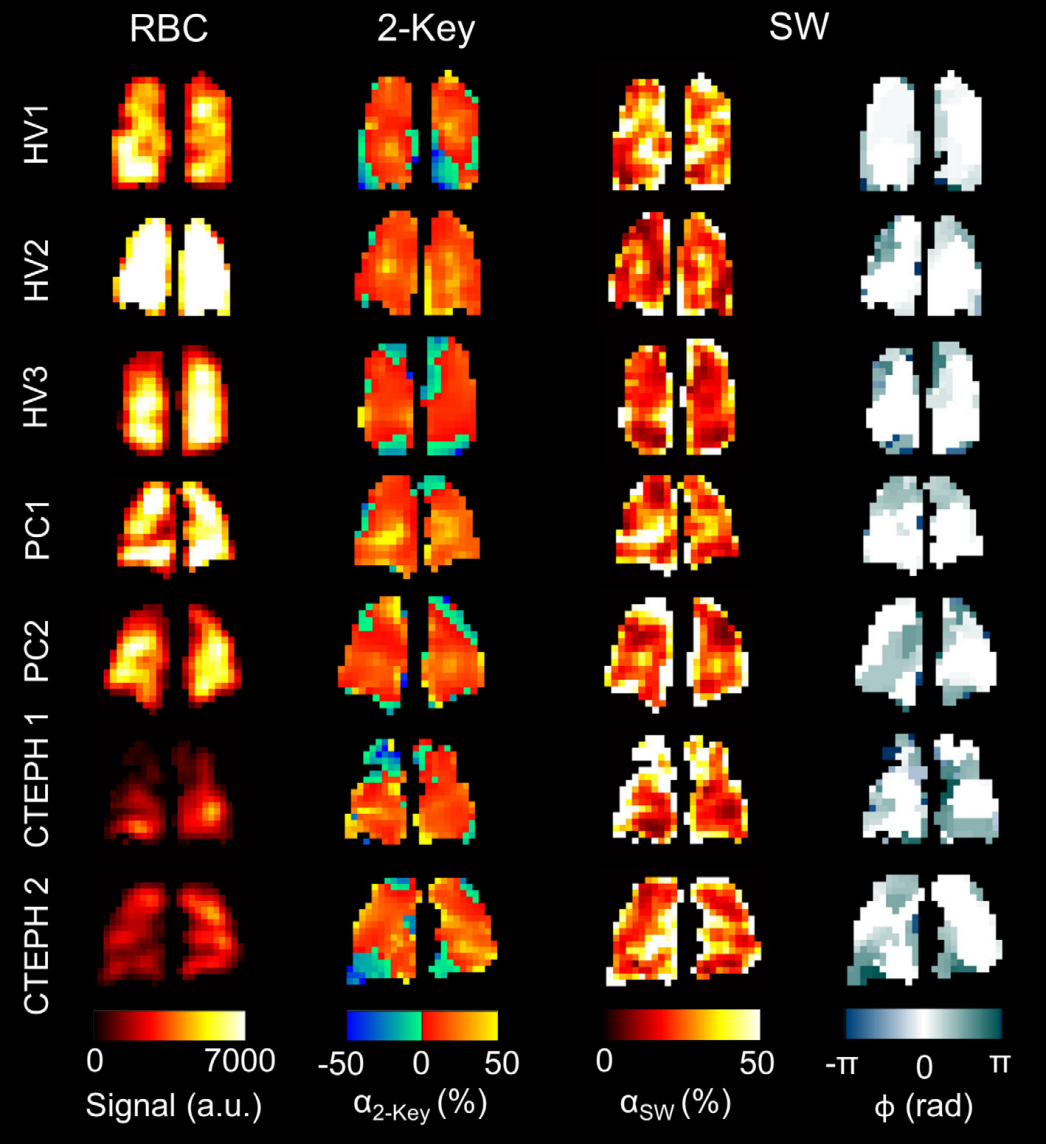
Maps of red blood cell (RBC) signal (reconstructed from all k‐space data), $\alpha_{2-\mathrm{Key}}$, $\alpha_{\mathrm{SW}}$ and ϕ, for 3 healthy volunteers (HV), 2 chronic thromboembolic pulmonary hypertension (CTEPH) subjects, and 2 post‐COVID‐19 (PC) residual lung abnormality subjects. Color map limits have been set to aid visualization. SW, sliding window.

The SW keyhole RBC signal revealed regional variations in oscillation phase as well as amplitude, whereas the Two‐Key method, by definition, only provides information on oscillation amplitude. Example $\alpha_{2-\mathrm{Key}}$, $\alpha_{\mathrm{SW}}$, and ϕ maps are shown in Figure 3. Table 1 provides the subject demographics and a summary of the RBC oscillation mapping results. On an individual subject basis, the $\alpha_{2-\mathrm{Key}}$ maps were normally distributed; hence, the mean was used, whereas the $\alpha_{\mathrm{SW}}$ and ϕ maps were not normally distributed, so the median value was used. Table 1 summarizes the intersubject means/medians.

**TABLE 1:** Subject demographics and intersubject α mapping results.

	Healthy	PC‐RLA	CTEPH
*n* (female)	28 (12)	5 (0)	4 (0)
Age (years)	38.8 ± 11.1	64.6 ± 9.7*	63.0 ± 7.3*
RBC:Gas	0.0040 ± 0.0011	0.0024 ± 0.0004*	0.0025 ± 0.0005*
RBC:M	0.41 (0.31–0.58)	0.23 ± 0.05*	0.25 ± 0.06*
$\alpha_{k0}$ (%)	15 (10–27)	25 ± 8*	15 ± 5
$\alpha_{2-\mathrm{Key}}$ (%)	14 ± 3	19 ± 6	10 ± 3
$\alpha_{\mathrm{SW}}$ (%)	29 ± 3	33 ± 4	33 ± 4
ϕ (rad)	0.27 ± 0.19	0.33 ± 0.19	0.24 ± 0.13
CV_2‐Key_	1.4 ± 0.3	1.2 ± 0.6	2.9 ± 0.9*
CV_SW_	0.66 ± 0.06	0.69 ± 0.07	0.76 ± 0.01*
CVϕ	0.85 ± 0.05	0.83 ± 0.04	0.92 ± 0.03*

*Note*: For the healthy subjects, normally distributed variables are given as mean ± SD, and nonnormally distributed variables are given as median (range). Normality was not tested for in the patient groups due to low numbers; for these, the mean ± SD is given. Asterisks signify a significant difference from the healthy group (*p* < 0.05).

Abbreviations: CTEPH, chronic thromboembolic pulmonary hypertension; CV, coefficient of variation; M, membrane; PC‐RLA, post‐COVID‐19 residual lung abnormality; RBC, red blood cells; SW, sliding window.

### 3.1 Oscillation mapping in healthy participants

For healthy volunteers, the means of the $\alpha_{2-\mathrm{Key}}$ maps showed significant correlation with the mean $\alpha_{k0}$ values (Spearman's ρ = 0.60; *p* = 0.001), but they were significantly different (*p* = 2 × 10^−4^). Median $\alpha_{\mathrm{SW}}$ was significantly correlated with both $\alpha_{k0}$ (ρ = 0.50, *p* = 0.008) and mean $\alpha_{2-\mathrm{Key}}$ (Pearson's r = 0.40, *p* = 0.04). The values of $\alpha_{\mathrm{SW}}$ and ϕ were both significantly negatively correlated with estimated heart rate (ρ = −0.43, *p* = 0.02; and ρ = −0.47, *p* = 0.01; respectively), but there was no significant correlation between heart rate and $\alpha_{k0}$ or $\alpha_{2-\mathrm{Key}}$. All of the $\alpha_{2-\mathrm{Key}}$ maps exhibited some regions of negative oscillation amplitude (RBC signal greater in the low‐key image than the high‐key image). These areas became positive in the $\alpha_{\mathrm{SW}}$ maps, and correspondingly, CV_SW_ was significantly lower than CV_2‐Key_ (*p* = 2 × 10^−12^).

**FIGURE 4:**
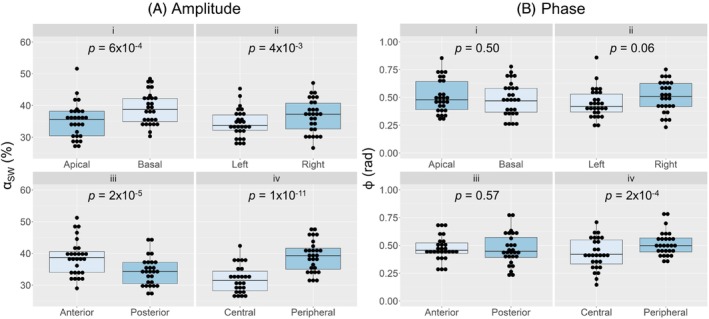
Comparison of $\alpha_{\mathrm{SW}}$ (A) and ϕ (B) among the apical/basal (i), left/right (ii), anterior/posterior (iii), and central/peripheral (iv) regions of the lung, with the corresponding *p*‐values from paired *t*‐tests and Wilcoxon signed‐rank tests. SW, sliding window.

The distribution of the healthy volunteer $\alpha_{\mathrm{SW}}$ values across all pixels and subjects was non‐Gaussian and had a median value of 29%, an interquartile range of 21%, and a range of (2–252)%. Several significant regional trends were identified for $\alpha_{\mathrm{SW}}$ (Figure 4A **)**. The value of $\alpha_{\mathrm{SW}}$ was observed to increase from the upper to lower, left to right, posterior to anterior, and central to peripheral lung. The value of ϕ was significantly greater in the peripheral lung than the central lung region, but no other significant regional trends were observed (Figure 4B).

**FIGURE 5:**
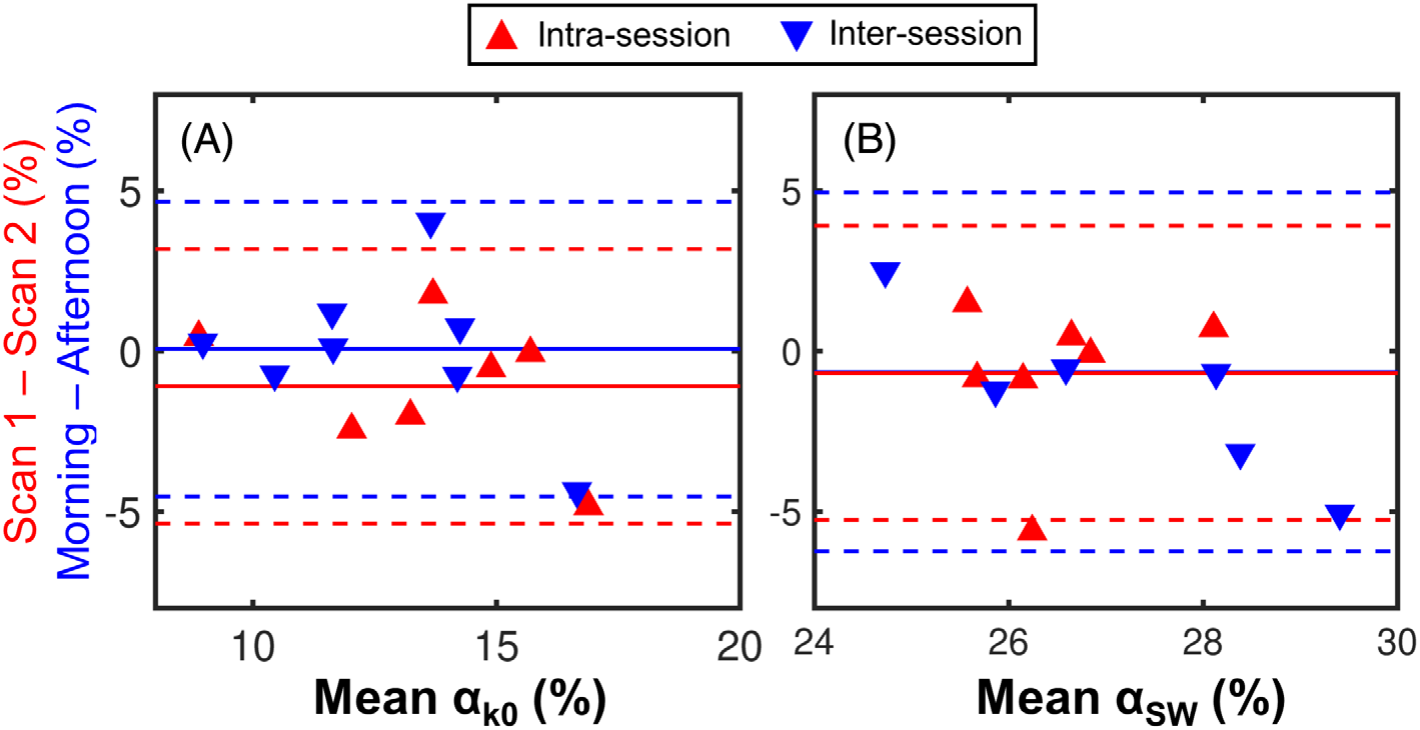
Bland–Altman plots of the intrasession and intersession repeatability of $\alpha_{k0}$ (A) and median $\alpha_{\mathrm{SW}}$ (B). The y‐axis shows the difference between the two oscillation amplitudes in units of percentage; (not percentage difference). SW, sliding window.

Same‐session and same‐day repeatability of $\alpha_{k0}$, mean $\alpha_{2-\mathrm{Key}}$, median $\alpha_{\mathrm{SW}}$, and median ϕ were investigated in 8 healthy subjects. The results are summarized in Table 2, and Bland–Altman plots for $\alpha_{k0}$ and $\alpha_{\mathrm{SW}}$ are shown in Figure 5. One of the intrasession data sets had to be discarded due to a technical fault at the scanner. The intrasession bias was smallest for $\alpha_{2-\mathrm{Key}}$, compared with $\alpha_{k0}$ and $\alpha_{\mathrm{SW}}$, whereas the intersession bias was smallest for $\alpha_{k0}$. To account for the increased value of $\alpha_{\mathrm{SW}}$ compared with $\alpha_{k0}$ and $\alpha_{2-\mathrm{Key}}$, the mean absolute percentage difference between α values was calculated. This was smallest for $\alpha_{\mathrm{SW}}$ both between sessions and within the same session, with an average variation of less than 10%. Mean CV across all three scans was also smallest for $\alpha_{\mathrm{SW}}$ (0.07 ± 0.04).

**TABLE 2:** Intrasession and intersession repeatability of $\alpha_{k0}$, mean $\alpha_{2-\mathrm{Key}}$, median $\alpha_{\mathrm{SW}}$, and median ϕ.

	Intrasession		Intersession		(3‐scan) CV
	Bias [LOA] (%)	% Difference (%)	Bias [LOA] (%)	% Difference (%)	
$\alpha_{k0}$	−1.09 [−5.37, 3.81]	13.3 ± 11.8	0.07 [−4.53, 4.66]	10.9 ± 10.9	0.11 ± 0.04
$\alpha_{2-\mathrm{Key}}$	−0.14 [−4.09, 3.82]	11.8 ± 9.1	−0.48 [−6.56, 5.60]	22.8 ± 12.2	0.16 ± 0.05
$\alpha_{\mathrm{SW}}$	−0.68 [−5.26, 3.91]	5.9 ± 8.2	−0.65 [−6.24, 4.95]	8.6 ± 6.3	0.07 ± 0.04
ϕ	−0.08 [−0.52, 0.37]	—	0.02 [−0.54, 0.57]	—	1.25 ± 1.95

*Note*: For the subject in whom one scan failed, data from a previous scan from 3 months prior were included to calculate the 3‐scan CV. Percentage difference was not calculated for ϕ due to values of zero.

Abbreviations: % Difference, mean absolute percentage difference; Bias, mean difference; CV, coefficient of variation across all three scans; LOA, limits of agreement/95% confidence interval.

### 3.2 Oscillation mapping in PC‐RLA and CTEPH participants

**FIGURE 6:**
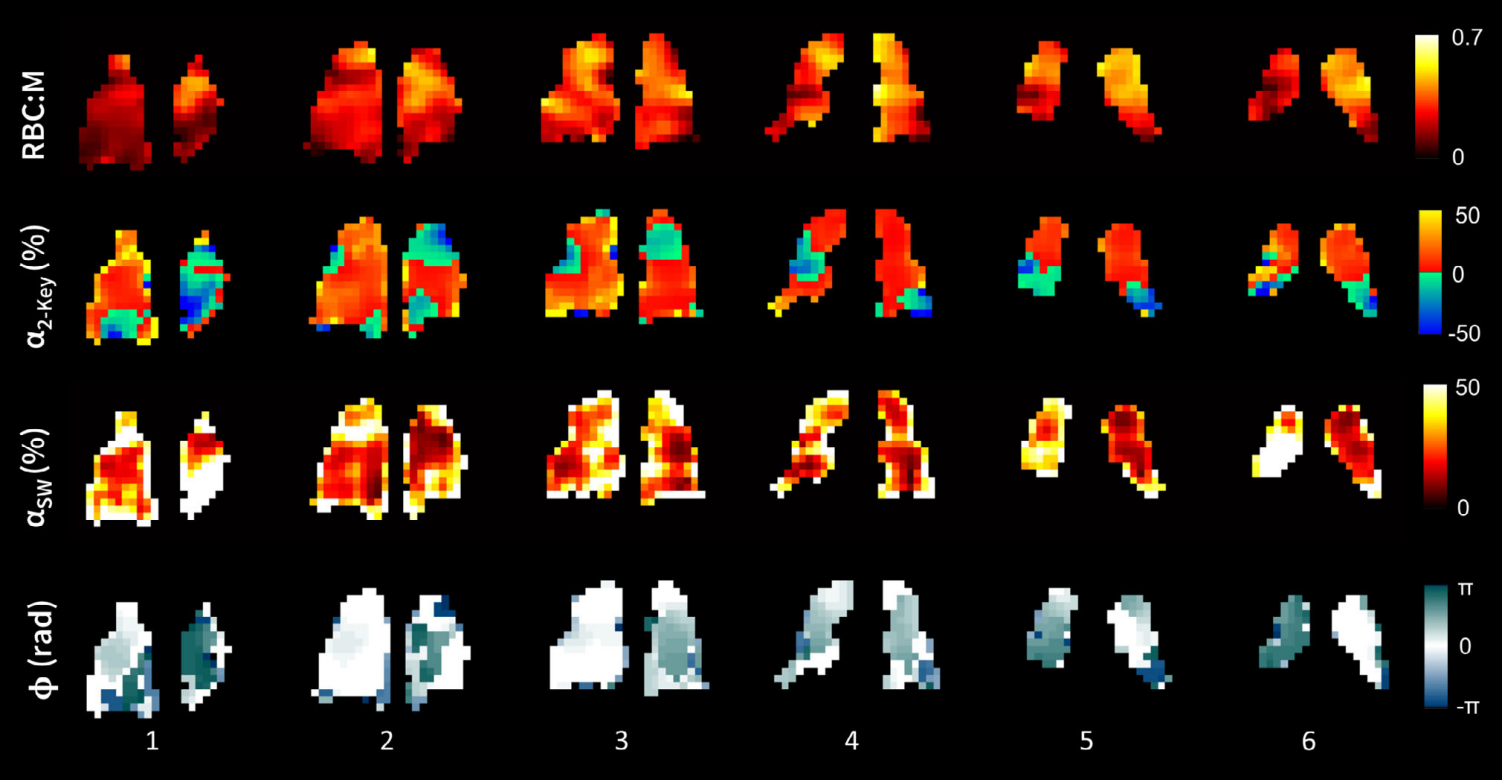
Maps of red blood cell (RBC): membrane (M) signal ratio, $\alpha_{2-\mathrm{Key}}$, $\alpha_{\mathrm{SW}}$, and ϕ for 1 patient with chronic thromboembolic pulmonary hypertension, shown for six slices of the lung, where 1–6 represents the posterior to anterior direction. This patient had a mean pulmonary arterial pressure of 58 mm Hg and a pulmonary vascular resistance of 10.1 WU. SW, sliding window.

The value of $\alpha_{k0}$ was significantly higher in the PC‐RLA patients than the healthy volunteers (*p* = 0.03), but no other significant differences were observed in the oscillation mapping metrics for this patient group. The oscillation amplitude and phase maps were qualitatively similar to those of the healthy volunteers and had similar CV values. In the CTEPH patients, $\alpha_{k0}$, $\alpha_{2-\mathrm{Key}}$, $\alpha_{\mathrm{SW}}$, and ϕ were similar to those of the healthy volunteers, but the $\alpha_{2-\mathrm{Key}}$, $\alpha_{\mathrm{SW}}$, and ϕ maps were more heterogeneous than those of the healthy volunteers, with significantly elevated CV values (*p* = 0.005, 0.02 and 0.03, respectively). Figure 6 shows the RBC:M, $\alpha_{2-\mathrm{Key}}$, $\alpha_{\mathrm{SW}}$, and ϕ maps for six slices of the lung for one of the CTEPH patients. There is substantial heterogeneity in the phase maps and in Slice 6, the left and right lung appear to be in antiphase with each other. Areas of RBC transfer defect correspond to regions of increased phase difference.

## 4 DISCUSSION

Oscillations of the ^129^Xe RBC signal originating from changes in the capillary blood volume over the cardiac cycle can be spatially resolved retrospectively from dissolved ^129^Xe spectroscopic imaging using keyhole reconstruction. Adapting the method of Niedbalski et al.^21^ for our multi‐echo spectroscopic imaging acquisition and data acquired therewith, we found a mean $\alpha_{2-\mathrm{Key}}$ of (14 ± 3)% in 28 healthy subjects. Mean $\alpha_{2-\mathrm{Key}}$ was correlated with mean $\alpha_{k0}$, although the values tended to be smaller. This may be because the low‐key and high‐key images were generated using approximately six projections either side of the extrema, whereas $\alpha_{k0}$ was calculated as the peak‐to‐peak amplitude. Our value was higher than the value of 8.7% found by Niedbalski et al.,^21^ although the mean $\alpha_{k0}$ was also higher for our subjects (15% when compared with 10%). The difference in $\alpha_{k0}$ may be because in the method of Niedbalski et al.,^21^ a sinusoidal fit was used to calculate the amplitude, whereas we used a peak detection algorithm, due to the underlying signal being non‐sinusoidal in shape.^40^ The latter method was recently shown to return higher α values using the same acquisition method as Niedbalski et al.^41^ In addition, the mean $\alpha_{2-\mathrm{Key}}$ is likely higher due to differences in the normalization methods used. We used a pixelwise normalization in which the pixelwise differences between $S_{\text{high}}$ and $S_{\mathrm{low}}$ were normalized by their mean values, whereas Niedbalski et al.^21^ normalized by the whole‐lung mean of the fully sampled RBC image. We chose our approach for two reasons: First, the RBC signal itself is spatially varying and subject to regional bias from B_1_ inhomogeneity; therefore, a regional normalization is more appropriate to distinguish trends in oscillation amplitude and phase from the mean RBC signal itself. Second, the fully sampled RBC image has higher signal compared with the keyhole images; therefore, normalization by the fully sampled image results in lower oscillation amplitudes. In other words, we chose the normalization image to undergo reconstruction with the same keyhole sampling pattern as the high and low images, so that the SNR was comparable.

We further adapted the previously published method to estimate and correct for regional phase differences of the RBC oscillation. By adopting a SW approach to select the k_0_ projections and repeating the keyhole reconstruction for each selection, we were able to resolve the pixelwise RBC signal evolution with time. The oscillation amplitudes were calculated individually for each pixel, without the assumption that the oscillation is in phase with the whole‐lung k_0_ oscillation. Using this method, areas of physiologically unrealistic negative oscillation amplitude in the Two‐Key maps became positive in the SW maps due to their regional phase correction. By converting the (key) index of the keyhole from which the first maximum originated for each pixel (*j*_max_) into phase, it was possible to create oscillation phase maps. Phase differences relative to the k_0_ oscillation occurred mostly in the peripheral lung; furthermore, these regions qualitatively correlated with areas of negative oscillation amplitude from the Two‐Key oscillation maps. This corroborates the hypothesis that negative oscillation amplitudes produced in the Two‐Key method are caused by phase differences. Phase differences are thought to originate from effects of the cardiac pulse wave, which will reach regions of the capillary bed at different times due to different distances from the heart. For a typical heart rate of 70 bpm, the maximum ϕ value of ± π corresponds to a delay of about 400 ms, which is of the order of the whole‐lung average conduction time of the cardiac pulse from the pulmonary valve to the capillary bed (120–180 ms).^42^, ^43^ Other regional variations in phase may result from cardiac pulse wave reflections due to impedance mismatch at bifurcations, ineffective vascular coupling related to focal lung or pulmonary vascular disease, or variations in blood flow velocity with vessel narrowing or change in vessel wall stiffness or compliance.

The replacement of negative oscillation values with positive values in the SW maps explains why the average regional $\alpha_{\mathrm{SW}}$ was higher than $\alpha_{k0}$ or $\alpha_{2-\mathrm{Key}}$. In addition, the distribution of $\alpha_{\mathrm{SW}}$ values was not normally distributed and was found to be positively skewed. The range of SW oscillation values was large, and some pixels at the periphery of the lung had oscillations of over 200%, which could be because of blurring or partial volume effects. These pixels tended to have very small RBC values (used as the denominator in the normalization process), and it is possible that the corresponding large oscillation values were partly due to noise.

Regional trends were observed in $\alpha_{\mathrm{SW}}$ in healthy volunteers. The anterior–posterior, center–peripheral, and left–right gradients reflect the reverse of the RBC signal trends, which is explained by the pixelwise normalization mentioned previously. No significant difference was found between the RBC signal at the base and the apex of the lung; however, a significant decrease was found in $\alpha_{\mathrm{SW}}$. Ventilation at the base of the lungs is increased in the supine position;^44^ in healthy volunteers with associated V/Q matching, there may also be an increase in perfusion, which may explain the reduced $\alpha_{\mathrm{SW}}$. To directly compare with the results of Niedbalski et al.,^21^ we also evaluated the regional trends of $\alpha_{2-\mathrm{Key}}$ when a normalization by the whole‐lung mean RBC signal was used. A significant increase was observed from the anterior to the posterior of the lung due to gravitational effects, similar to that work. No significant changes were found between the base and apex, or the left and right lung, but a significant decrease was found between the core and peripheral lung, also in agreement with Niedbalski et al.^21^

The value of $\alpha_{\mathrm{SW}}$ exhibited a moderate (ρ = −0.43), significant (*p* = 0.02), negative correlation with the (RBC signal–derived) heart rate for the healthy volunteers. This may be interpreted by considering the effect of the heart rate on pulmonary blood flow. With increased heart rate, the heart spends relatively less time in diastole per beat if the stroke volume, pulmonary vascular resistance, and compliance remain the same.^45^ A higher blood flow is maintained throughout the cardiac cycle, and blood flow pulsatility is reduced. This effect is propagated to the pulmonary capillaries; therefore, the relative change in capillary blood volume, and hence $\alpha_{\mathrm{SW}}$, is decreased. Significant correlations were not seen with heart rate and either $\alpha_{k0}$ or $\alpha_{2-\mathrm{Key}}$. As far as we are aware, a relationship between ^129^Xe RBC oscillation amplitude and heart rate has not been reported previously; this warrants further investigation in healthy volunteers and patient groups.

In a subgroup of 8 healthy volunteers, we demonstrated that RBC oscillation phase mapping is repeatable between scans, with a smaller intrasession bias (−0.68%) for $\alpha_{\mathrm{SW}}$ than that of $\alpha_{k0}$ (−1.09%). The $\alpha_{\mathrm{SW}}$ bias between separate examinations on the same day (−0.65%) was similar to the intrasession bias but greater than that of $\alpha_{k0}$ and $\alpha_{2-\mathrm{Key}}$. However, $\alpha_{\mathrm{SW}}$ is larger on average than $\alpha_{k0}$ and $\alpha_{2-\mathrm{Key}}$, and when the mean absolute percentage difference and CV across all three scans were compared, these were smallest for $\alpha_{\mathrm{SW}}$ (8.6% and 0.07). This may be because $\alpha_{\mathrm{SW}}$ is independent of phase, so one source of variation that might occur between scans was removed. Testing with a larger group of subjects—including patients with a wider range of pulmonary diseases—is required to fully assess the repeatability of this method.

To demonstrate the potential application of this method to lung disease groups, RBC oscillation mapping was tested in 4 patients with CTEPH and 5 patients following hospitalization with COVID‐19 pneumonia who had ILD‐like RLA. The focus of the present paper is methodology of the RBC oscillation phase‐mapping technique rather than assessment of differences between patients with different clinical diseases versus volunteers and their pathophysiological interpretation. Nevertheless, our preliminary observations show that $\alpha_{k0}$, $\alpha_{2-\mathrm{Key}}$, $\alpha_{\mathrm{SW}}$, and ϕ appear elevated in the PC‐RLA patients when compared with the healthy volunteers, although only the increase in $\alpha_{k0}$ was significant. There is a lack of literature on α in PC patients, but it is reported to be increased in patients with IPF.^6^, ^9^, ^17^ Areas of increased α and ϕ in the PC‐RLA patients may therefore reflect regions of ILD‐like inflammation or fibrosis (see Figure S3 for an example comparison to CT).

Although mean $\alpha_{k0}$ for the CTEPH group was similar to that of the healthy group, the oscillation amplitude and phase maps both revealed significant heterogeneity. This demonstrates the potential of oscillation mapping to identify microvascular abnormalities, which might otherwise be lost in the whole‐lung average oscillation amplitude. The ability of the SW keyhole reconstruction to estimate RBC oscillation phase may be particularly useful in this patient group. CTEPH is characterized by vascular occlusion following pulmonary embolism, along with microvascular disease.^46^ Therefore, the observed phase differences in the ^129^Xe RBC signal oscillations may represent a hemodynamic response at the capillary level to impedance to flow from vascular thrombi in the larger vasculature. The sensitivity of ^129^Xe RBC oscillation mapping to pulmonary capillary hemodynamics may provide a useful tool for identifying arterial remodeling and indicating the extent of microvascular disease. Estimation of oscillation phase also offers a parallel to other measurements of the pulse wave, such as the pulmonary pulse wave transit time from PREFUL (phase‐resolved functional lung imaging), which has been found to be longer in CTEPH patients.^47^

### 4.1 Limitations

The primary limitation of this method is the requirement for high‐SNR RBC images and clear cardiogenic RBC signal oscillations. This may limit the application of the method to lung disease patients who struggle to inhale the full ^129^Xe dose or complete the 14‐s breath hold due to their symptoms, or who might have inherently lower RBC signal due to reduced gas transfer. The minimum SNR condition was not met in a high proportion of the healthy volunteer data inspected, which was primarily due to a temporary dip in polarizer/RF coil performance.

Another limitation is that the frequency and waveform of the RBC k_0_ oscillation were not constant over the breath hold, because of signal noise and heart rate changes. As *j* approaches *N*, different sections of each cardiac cycle waveform are sampled, because the projections selected by the SW become “out of step” with each other. This effect can be seen in the projections chosen for the *j* = *N* keyhole in Figure 2A. Decreasing the TR of the imaging sequence, while maintaining the TR_90,equiv_ with a corresponding change in flip angle,^34^ may help to mitigate this effect by reducing the duration of the breath hold. A shorter TR would also be advantageous for increasing the temporal resolution of the k_0_ signal, but this might be unattainable for our four‐echo sequence.

The small number of lung disease patients included in this methodological work reduced the statistical power of the comparisons between groups and therefore increased the probability of a false negative. Future work will include evaluating our method in increased subject numbers, including additional pulmonary hypertension subtypes. It is challenging to validate our RBC oscillation phase‐mapping method because there is a lack of well‐established imaging methods to quantify the function of the pulmonary microvasculature, and more work is needed to compare RBC oscillation phase mapping to techniques such as quantitative dynamic contrast‐enhanced MRI. Comparisons with metrics of the pulse wave from PREFUL MRI may also be insightful for both healthy subjects and those with pulmonary disease.

## 5 CONCLUSION

Cardiogenic oscillations of the ^129^Xe RBC signal can be mapped retrospectively from multipoint 3D radial dissolved spectroscopic imaging using a keyhole reconstruction scheme. This work builds on previous methodology by sampling regional phase differences in RBC oscillation using a SW keyhole reconstruction. This approach allows for the oscillation phase to be regionally estimated, which may provide a means to detect the effects of the cardiac pulse wave in the pulmonary microvasculature and its alteration in cardiopulmonary disease.

## Supporting information


**Figure S1.** To isolate the ^129^Xe red blood cell (RBC) signal oscillations for oscillation mapping, we start with the raw membrane (A) and RBC (B) data from the center of radial k‐space (k_0_). (C) The membrane k_0_ signal is normalized by its mean and then fit to a biexponential decay model. (D) The RBC k_0_ signal is normalized by its mean, then corrected for RF and T_1_ depolarization effects by dividing by the membrane fit. (E) A band‐pass filter of 0.5–2.5 Hz is used to smooth and further detrend the RBC k_0_ oscillations. (F) A peak detect algorithm is used to identify the maxima and minima of the oscillations. These are used to find the whole‐lung oscillation amplitude, $\alpha_{k0}$, and to create the keyhole k‐space.
**Figure S2.** Schematic of the calculation of α from k_0_, the “Two‐Key” method, and the “Sliding Window”/red blood cell (RBC) oscillation phase mapping method.
**Figure S3.** (A) CT image of a central lung slice for a post‐COVID‐19 patient with residual lung abnormalities (PC‐RLA) and the red blood cell (RBC) oscillation maps for a central lung slice: (B) phase map, (C) “Two‐Key” method oscillation amplitude map, and (D) sliding window method oscillation amplitude map. The CT image shows increased opacity in the upper left lung, which qualitatively corresponds to a region of increased phase difference in (B).
**Table S1.** Clinical information for the 4 patients with chronic thromboembolic pulmonary hypertension (CTEPH).

## Data Availability

The open‐source code and an example data set to perform SW oscillation phase mapping can be found at https://github.com/POLARIS‐Sheffield/rbc‐phase‐map.
