# Supplementary material for: Mapping the amplitude and phase of dissolved 129Xe red blood cell signal oscillations with keyhole spectroscopic lung imaging
Source: Magn Reson Med. 2024 Oct 18;93(2):584–96. doi: 10.1002/mrm.30296 (PMC11604899; doi:10.1002/mrm.30296)
Supplement: Supplementary file 1 — Figure S1. To isolate the 129Xe red blood cell (RBC) signal oscillations for oscillation mapping, we start with the raw membrane (A) and RBC (B) data from the center of radial k‐space (k0). (C) The membrane k0 signal is normalized by its mean and then fit to a biexponential decay model. (D) The RBC k0 signal is normalized by its mean, then corrected for RF and T1 depolarization effects by dividing by the membrane fit. (E) A band‐pass filter of 0.5–2.5 Hz is used to smooth and further detrend the RBC k0 oscillations. (F) A peak detect algorithm is used to identify the maxima and minima of the oscillations. These are used to find the whole‐lung oscillation amplitude, αk0, and to create the keyhole k‐space. Figure S2. Schematic of the calculation of α from k0, the “Two‐Key” method, and the “Sliding Window”/red blood cell (RBC) oscillation phase mapping method. Figure S3. (A) CT image of a central lung slice for a post‐COVID‐19 patient with residual lung abnormalities (PC‐RLA) and the red blood cell (RBC) oscillation maps for a central lung slice: (B) phase map, (C) “Two‐Key” method oscillation amplitude map, and (D) sliding window method oscillation amplitude map. The CT image shows increased opacity in the upper left lung, which qualitatively corresponds to a region of increased phase difference in (B). Table S1. Clinical information for the 4 patients with chronic thromboembolic pulmonary hypertension (CTEPH). [file MRM-93-584-s001.docx]

**Supporting Information for ‘Mapping the amplitude and phase of dissolved ^129^Xe red blood cell signal oscillations with keyhole spectroscopic lung imaging’, J.H. Pilgrim-Morris et al**


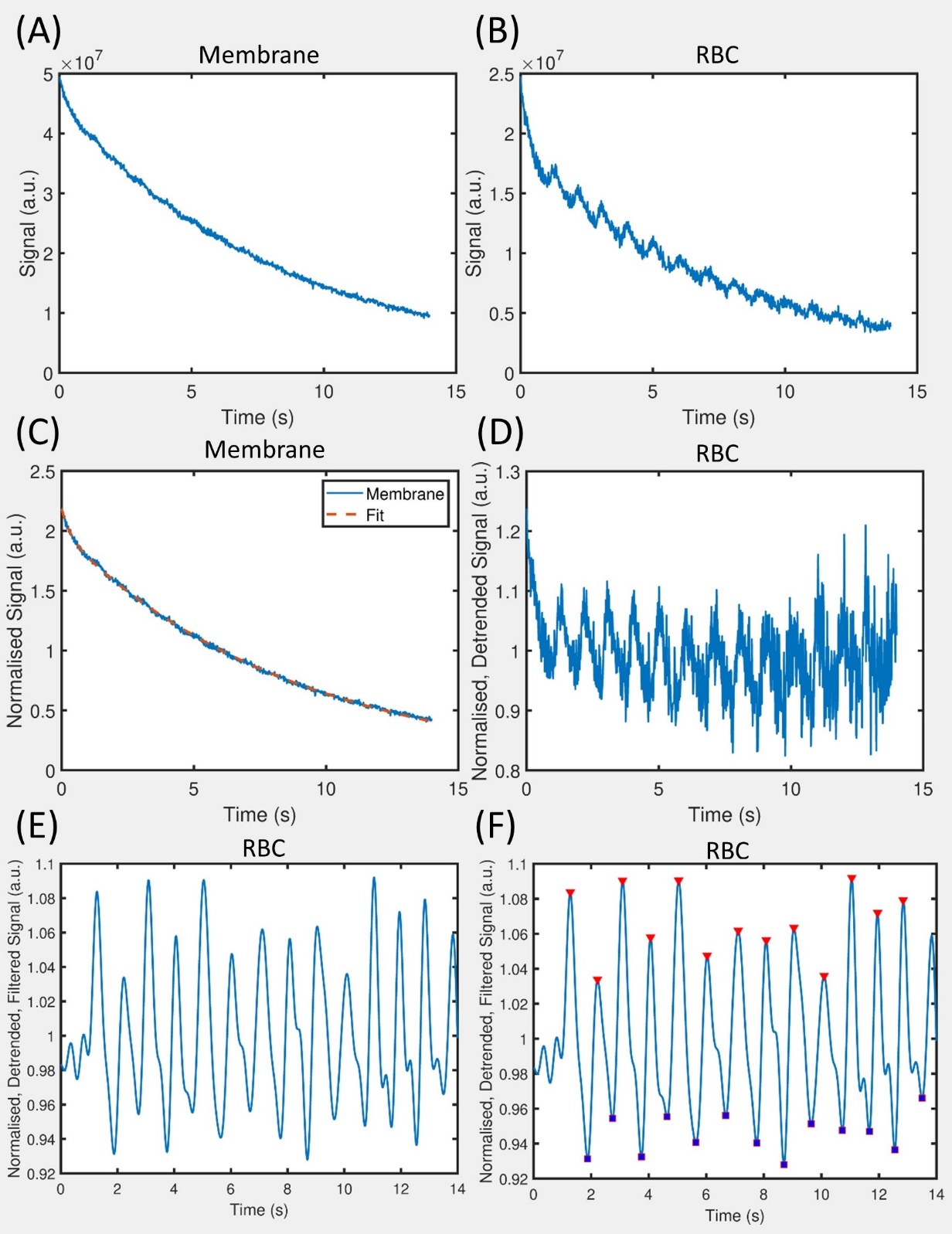


Figure S1: To isolate the ^129^Xe RBC signal oscillations for oscillation mapping, we start with the raw (A) membrane and (B) RBC data from the center of radial k-space (k_0_). (C) The membrane k_0_ signal is normalized by its mean and then fit to a biexponential decay model. (D) The RBC k_0_ signal is normalized by its mean, then corrected for RF and T_1_ depolarization effects by dividing by the membrane fit. (E) A band-pass filter of 0.5 - 2.5 Hz is used to smooth and further detrend the RBC k_0_ oscillations. (F) A peak detect algorithm is used to identify the maxima and minima of the oscillations. These are used to find the whole-lung oscillation amplitude, $\alpha_{k0}$ and to create the keyhole k-space.


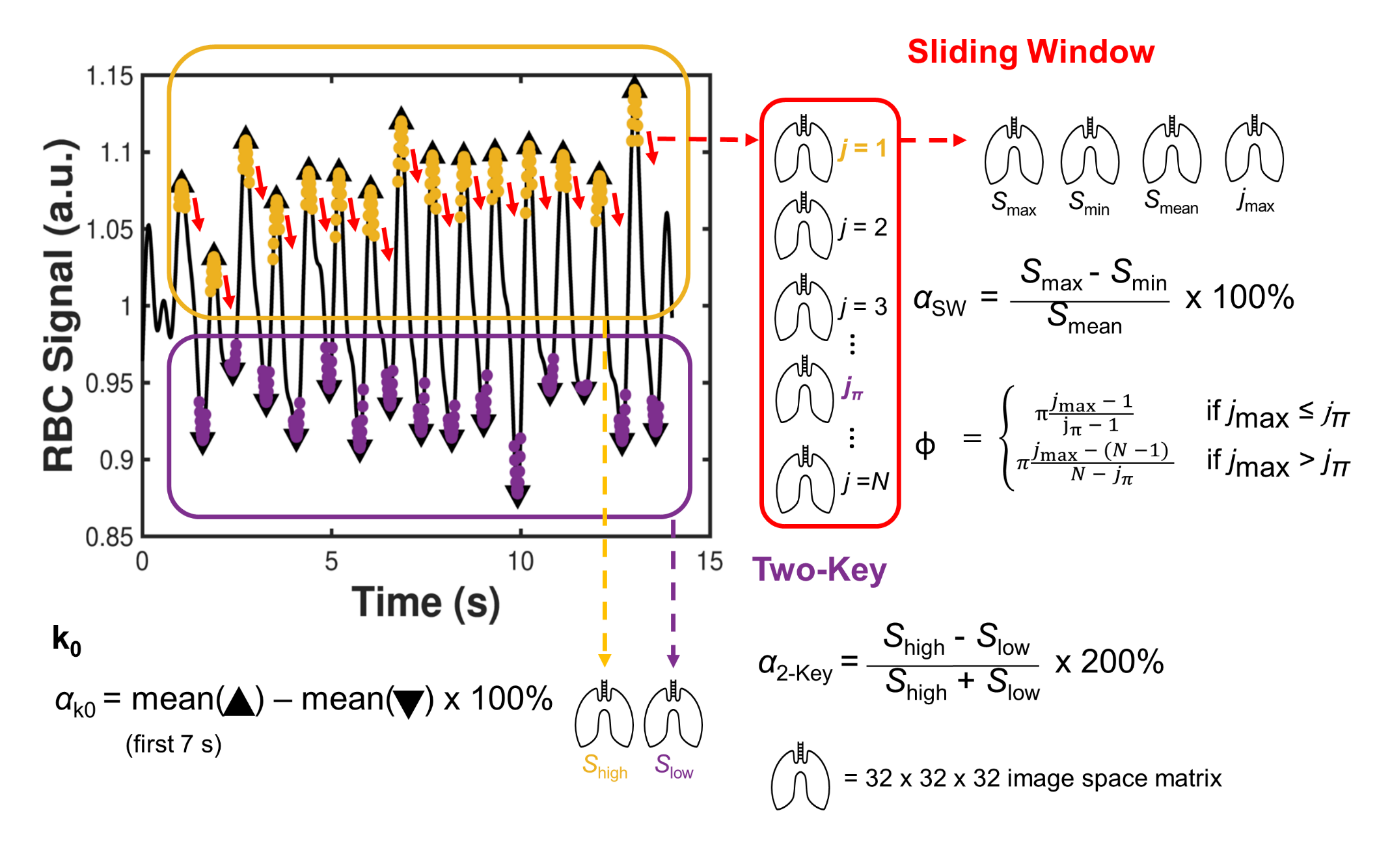


Figure S2: Schematic of the calculation of $\alpha$ from k_0_, the “Two-Key” method and the “Sliding Window”/RBC oscillation phase mapping method.

k_0_: $\alpha_{k0}$ is the difference between the mean of the maxima and the mean of the minima found from the k_0_ RBC signal from the first ~7 s.

Two-Key: $\alpha_{2-Key}$ is the difference between the reconstructed high-key and low-key images, normalized by the mean of the high- and low-key images.

Sliding Window: the k_0_ projections chosen for the first key (*j* = 1) are the same as those chosen for the high key (yellow points) in the Two-Key method. N keyhole reconstructions are carried out, moving the projections chosen for the key forward by one each time. This results in N keyhole images. The pixelwise minimum, maximum, and mean signals are found ($S_{\min}$, $S_{\max}$ and $S_{\mathrm{mean}}$), as well as the index of the keyhole image which corresponded to $S_{\max}$ (*j*_max_). $\alpha_{\mathrm{SW}}$ is found from the difference between $S_{\max}$ and $S_{\min}$, normalized by $S_{\mathrm{mean}}$. To find $\phi$ from *j*_max_, first we identify *j_π_*; the index of the keyhole image for which the k_0_ projections chosen for the key are approximately π out of phase with the *j* = 1 projections, i.e. most evenly distributed around the k_0_ minima. Once this is known, a simple piecewise transformation is applied to convert *j*_max_ to a discrete phase, which is defined relative to the phase of the k_0_ oscillation, such that:

*j*_max_ = 1 corresponds to $\phi$ = 0,

*j*_max_ = 2 corresponds to $\phi=$ $d\phi$_1_,

*j*_max_ = 3 corresponds to $\phi=2d\phi$_1_,

*j*_max_ *= j_π_* corresponds to $\phi$ = π

*j*_max_ *=* N - 1 corresponds to $\phi=-2d\phi$_2_,

*j*_max_ *=* N corresponds to $\phi=-d\phi$_2_,

where $d\phi$_1_ and $d\phi$_2_ are discrete phases, which depend on the magnitude of *j_π_* and (N *- j_π_)* respectively.


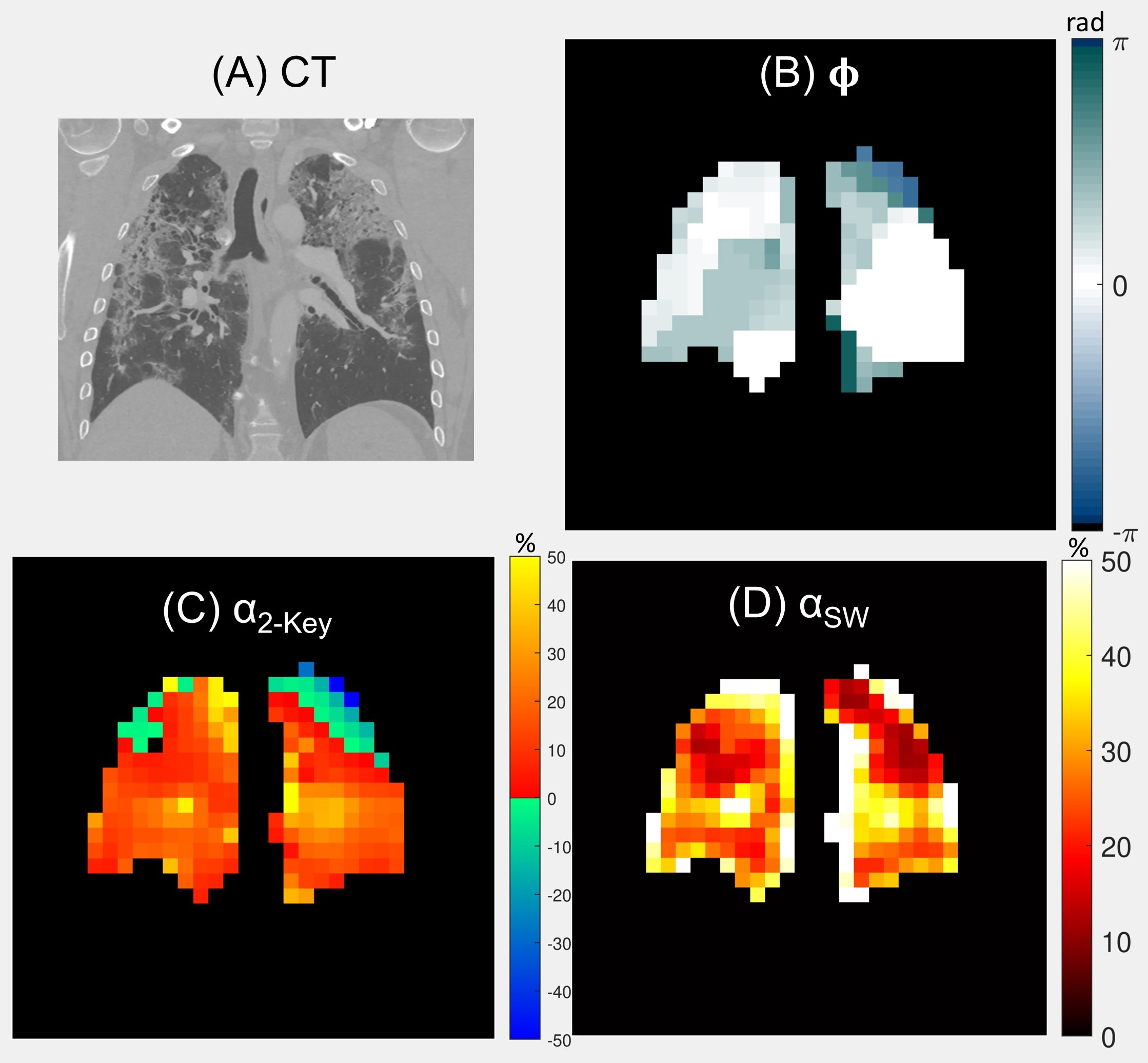


Figure S3: (A) CT image of a central lung slice for a post-COVID-19 patient with residual lung abnormalities (PC-RLA) and the RBC oscillation maps for a central lung slice: (B) phase map (C) Two-Key method oscillation amplitude map (D) Sliding window method oscillation amplitude map. The CT image shows increased opacity in the upper left lung, which qualitatively corresponds to a region of increased phase difference in (B).

Table S1: Clinical information for the four patients with CTEPH.

|  | CTEPH001 | CTEPH002 | CTEPH003 | CTEPH004 |
| --- | --- | --- | --- | --- |
| Age | 54 | 71 | 66 | 61 |
| Sex | Male | Male | Male | Male |
| WHO functional class | II | III/IV | III | Unknown |
| Comorbidities | COPD | Type 1 respiratory failure | Obstructive sleep apnea | Pneumonia (January 2024) |
| Prior PEA/BPA? | PEA | No | BPA | No |
| mPAP (mm Hg) | 32 | 58 | 54 | 36 |
| PVR (WU) | 3.2 | 10.1 | 10.6 | 4.1 |

*Abbreviations: CTEPH = chronic thromboembolic pulmonary hypertension, COPD = chronic obstructive pulmonary disease, PEA = pulmonary endarterectomy, BPA = balloon pulmonary angioplasty, mPAP = mean pulmonary arterial pressure, PVR = pulmonary vascular resistance (Wood’s units)*
